# Weight Stigmatization and Binge Eating in Asian Americans with Overweight and Obesity

**DOI:** 10.3390/ijerph17124319

**Published:** 2020-06-17

**Authors:** Ya-Ke Wu, Diane C. Berry, Todd A. Schwartz

**Affiliations:** 1 School of Nursing, The University of North Carolina at Chapel Hill, Chapel Hill, NC 27599-7460, USA; dberry@email.unc.edu (D.C.B.); tschwart@email.unc.edu (T.A.S.); 2 Department of Biostatistics, Gillings School of Global Public Health, The University of North Carolina at Chapel Hill, Chapel Hill, NC 27599-7460, USA

**Keywords:** weight stigma, binge eating, Asian Americans, obesity

## Abstract

Weight stigma and binge eating have been found to be associated in Western populations; however, this relationship is understudied among Asian Americans. The aims of the study were to (1) investigate the prevalence of binge eating and its relationship with experienced weight stigma in higher-weight Asian Americans, and (2) examine whether the level of acculturation moderates this relationship. Data were collected from a cross-sectional study with 166 higher-weight Asian American adults living in North Carolina, United States. Demographic data, the frequency of experiencing weight stigma, the severity of binge eating, the levels of acculturation, the perceived racism against Asians, and perceived stress were assessed via self-reported questionnaires. The results indicated that experienced weight stigma was a significant independent predictor over and above the effects of other stressors, such as racism and general stress. The level of acculturation did not influence the relationship between the experienced weight stigma and binge eating after adjusting for relevant covariates. Our findings contribute to the limited literature examining weight stigma and binge eating among Asian American populations, highlighting that higher levels of experienced weight stigma are associated with a greater degree of binge eating.

## 1. Introduction

Weight stigma, a pervasive social problem for individuals with higher body weight, has been widely investigated in Western populations. Individuals with a higher body weight often experience situations such as being stereotyped as unintelligent, lazy, greedy for food, and lacking self-discipline, as well as receiving negative criticisms, physical harassment, and unfair treatment because of their weight [1]. These experiences may be considered weight stigma, and individuals who experience stress from weight stigma may ultimately develop self-blame and shame [2]. Previous research has shown that weight stigma is associated with poorer mental (e.g., depressive symptoms and anxiety disorders) and physical (e.g., metabolic dysregulation and inflammation) health outcomes [3,4,5]. Racial differences in weight-related concerns and teasing have been documented in previous studies on youths [6,7]. Asian American girls reported higher percentages of perceiving themselves as overweight, with lower levels of body satisfaction compared with other races such as non-Hispanic white, African American, and Hispanic-white [6]. Asian-American, Hispanic, and mixed-race girls reported a higher prevalence of weight teasing by family members compared with non-Hispanic white girls [7]. Although the issue of weight stigma has received increased attention in recent years, the topic of weight stigma in Asian Americans remains understudied. Exploring the impact of weight stigma on Asian Americans’ health can fill the gap in the weight stigma literature in underrepresented populations and inform the development of interventions targeting the specific needs of Asian Americans who experience weight stigma.

Multiple studies have indicated that the experience of weight stigma is significantly associated with binge eating [8,9,10,11,12]. Binge eating is defined as recurrent episodes of eating an unusually large amount of food in a short period of time, with an associated loss of control over-eating during the episode [13]. The diagnostic criteria for binge-eating disorder (BED) include recurrent episodes of binge eating (i.e., at least 1 per week for 3 months), eating or emotional disturbances (e.g., eating until feeling uncomfortably full and feeling disgusted or guilty after overeating), and not associated with regular use of inappropriate compensatory behavior (e.g., purging or excessive exercise) [13]. The theory of stress-induced food-reward behavior may be a possible explanation of the relationship between weight stigma and binge eating [14,15]. Experimental studies suggest causation between weight stigma and eating in excess of hunger [16], such that overweight women who watched a movie that contained weight stigmatizing messages ate three times more calories compared to overweight women who watched a movie without weight stigmatizing messages [17]. Previous studies have characterized weight stigma as a chronic psychological stressor, and binge eating has been examined as an ineffective coping strategy to escape the stress caused by weight stigma [2,10,18]. Binge eating with palatable food (i.e., tasty, high-calorie foods containing high amounts of sugars, fats, and carbohydrates) after experiencing stress may be driven by reward rather than metabolic need [19]. Palatable food intake relieves negative emotions by activating neural reward circuitry and eliciting dopamine release from the amygdala and nucleus accumbens and dampening the signs of stress following exposure to acute or chronic stressors [20,21]. A few preliminary studies showed that food craving might be ethnicity-dependent. For example, Asian American college students with a particular genotype of a dopamine-resistant receptor polymorphism, DRD2 A1, an allele of a dopamine receptor gene that is associated with food addiction, had higher food cravings for carbohydrates and fast food than those without the allele [22]. Japanese female college students also reported higher food cravings for carbohydrates such as rice and onigiri compared with food cravings for foods high in fats such as fried chicken and sausage [23]. Although the prevalence of BED diagnoses are similar across Hispanic-whites, Asians, African Americans, and non-Hispanic whites living in the United States (US) [24], Asian Americans report a higher prevalence of binge eating symptoms compared with non-Hispanic whites [25]. Yet, compared with non-Hispanic whites, Asian Americans and other underrepresented groups who have disordered eating are underdiagnosed, undertreated, and report a lower intention to seek professional help [25,26]. Whether or not the diagnostic criteria for BED are met, the negative impact of binge eating on health outcomes exist, such as the increased risk for developing obesity and type 2 diabetes as well as comorbid psychiatric disorders like anxiety and mood disorders, resulting in an impaired mental health-related quality of life [27]. It is important to note that not all high-weight individuals binge eat [28], and weight stigma experiences can happen in individuals of all body sizes. Only 36–42% of individuals with a lifetime BED diagnosis are obese [28], indicating that binge eating also occurs in individuals without excess weight. Although the association between weight stigma and binge eating are well established, limited research has been dedicated to these topics exclusively in Asian Americans. It is important to understand these issues in Asian Americans to provide information to reduce health disparities among Asian Americans.

When examining weight stigma in Asian Americans, it is necessary to consider acculturation status, which may relate to the change in eating habits for Asian Americans. Acculturation is a process through which an individual from one culture comes to adopt the beliefs, behaviors, practices, and values of another culture [29]. Asian immigrants start the process of acculturation when they migrate from Asia to the US [29]. One of the effects of acculturation is that the environment and social gatherings with individuals encourage Asian immigrants to try the American lifestyle, including the traditional American diet [30]. A previous study showed that acculturation to the US was significantly associated with a higher frequency of fast-food consumption among Asian American adolescents, and this result was consistent across sexes [31]. Another study reported that Asian Americans might consume more high-fat, high-caloric traditional American foods when their American identity was challenged (i.e., being asked if they were Americans) [32]. The two previously described studies indicate that immigration to the US may provide an environment in which it is easy to consume fast foods and could increase the tendency for Asian Americans to eat more palatable foods, a type of food that binge eaters often consume to relieve negative emotions from stress [21]. Thus, immigrants with a high acculturation (i.e., more adapted to American culture) will likely be associated with a greater consumption of the highly palatable American diet and this will possibly contribute to weight gain and exposure to weight stigma situations. Additionally, the influences of sociocultural factors from American society may re-shape the body size preference of immigrants in the US due to the strong preference for the thin ideal in American culture [33]. For example, Latinx, Hmong, and Somali adolescents reported more body dissatisfaction, depressive symptoms, lower self-esteem, and more unhealthy weight control behaviors compared with non-Hispanic white adolescents, indicating that youths from immigrant communities may desire to be thinner and use unhealthy means of weight control, such as dietary restraint for weight loss [33]. Thus, acculturation is more likely to be associated with dietary restraint, and dieting for weight loss is known to be a risk factor for binge eating as well as a coping response to weight stigma [27,34]. Hence, we were interested in how the level of acculturation influences (e.g., mitigates or exacerbates) the association between weight stigma and binge eating in Asian Americans. 

The purpose of this study was to examine the relationship among the self-reported experienced weight stigma, binge eating, and acculturation among higher-weight Asian Americans (≥18 years of age) living in North Carolina (NC), the US using a cross-sectional study design. The specific aims were to (1) investigate the prevalence of binge eating, (2) examine the relationship between experienced weight stigma and binge eating among higher-weight Asian American adults, and (3) explore whether the level of acculturation moderates this relationship. The prevalence of experienced weight stigma in our sample has been previously published [35]. We also measured and considered the levels of perceived racism against Asian Americans and perceived stress as potentially confounding variables, because life-related stressful events may affect the severity of binge eating [36]. We hypothesized a significant positive association between experienced weight stigma and binge eating after statistically adjusting for confounding variables. We also hypothesized that the level of acculturation would significantly moderate the relationship between the experienced weight stigma and binge eating after statistically adjusting for confounding variables, meaning specifically that experienced weight stigma would be associated with higher levels of binge eating in more-acculturated individuals compared with less-acculturated individuals.

## 2. Materials and Methods 

### 2.1. Participants

A cross-sectional study from April 2016 to November 2016 was conducted in NC. The research materials supporting this publication can be accessed by contacting Dr. Ya-Ke Wu. This study was approved by the Institutional Review Board for the Protection of Human Subjects at the University of North Carolina at Chapel Hill. All the participants provided informed consent, and anonymity was preserved. The inclusion criteria were (a) US-born or foreign-born Asian Americans living in NC; (b) being ≥ 18 years of age; (c) the ability to read and write in English at a sixth-grade level or above [37]; and (d) having a body mass index (BMI) ≥ 23 kg/m^2^. Based on the suggestions of the World Health Organization Expert Consultation for the appropriate BMI for Asian populations for public health action, we employed the BMI cut-off points of 23 kg/m^2^ to 27.5 kg/m^2^ for “overweight” and greater than 27.5 kg/m^2^ for “obese” [38]. We understand that the World Health Organization (WHO) does not necessarily support the use of the BMI categorization used in the present study, and the WHO’s recommendations of the appropriate BMI cut-off points for Asians were based on the findings of the Asian population and not Asian Americans. Although the sample of our study is Asian Americans, the average (±standard deviation (SD)) duration for living in Asia was 26.6 ± 9.4 years in our sample, and the majority of our participants (94%) reported themselves as first-generation immigrants (i.e., a participant was born in Asia or country other than the United States). Additionally, previous weight loss research conducted in Asia used the similar low BMI cut-off points of “overweight” and “obesity” as our study [39,40]. Therefore, we decided to use the WHO [38] criteria in the present study. The participants’ exclusion criteria were having a mental illness (e.g., schizophrenia disorders) or cognitive limitations, which would have made it challenging to complete the surveys [41,42]. 

### 2.2. Sample Size Estimation

G*Power 3 is a software developed by the G*Power Team at Heinrich-Heine-Universität Düsseldorf, Düsseldorf, Germany, and was used for the power analysis of the present study [43]. The sample size was determined based on a hierarchical regression model of binge eating from a previous study by the first author in a Taiwanese sample with an effect size of 0.12 (i.e., change in *R*^2^ by adding both weight stigma and BMI in the hierarchical regression model) to achieve a statistical power of 0.80 with a two-sided α set at 0.05 [44]. The number of explanatory factors was set at 8 based on the number of independent and major confounding variables in the present study. The results of the power analysis indicated that 140 participants were required to achieve this level of power. The percentage of participants who may not complete data collection (and, hence, were excluded from analysis) was projected to be 20% [45]; therefore, 168 participants were needed to be enrolled in the study. One hundred sixty-eight participants were recruited; however, two participants did not complete the surveys and were excluded from the present study. Thus, valid and complete data were available and analyzed for 166 participants. 

### 2.3. Measures

#### 2.3.1. Demographic Variables

All the participants were asked to report their age, sex, education level, and the number of years they had lived in the US and Asia.

#### 2.3.2. Anthropometric Measurements

The height and weight of all the participants were measured. All the participants were dressed in light-weight indoor clothes without socks and shoes when their heights and weights were measured. The height was measured twice with a portable Martin stadiometer and was averaged and recorded to the nearest 0.5 cm. The weight was also measured twice with a portable digital scale and was averaged and recorded to the nearest 0.1 kg. The BMI was calculated by entering the averaged weight and height data into a BMI computerized formula: BMI = weight (kg)/height^2^ (m^2^). 

#### 2.3.3. Experienced Weight Stigma 

The participants’ experiences of weight stigma were measured using the Stigmatizing Situations Inventory [46]. The inventory contains 50 items to measure the self-reported frequency of experiencing weight stigma situations encountered by individuals. A 4-point scale was used in the present study (0 = never experienced the weight stigma situation in question; 1 = experienced one instance; 2 = experienced more than one instance; 3 = experienced multiple instances) [34]. The overall score of the inventory was computed by adding all the questions and dividing by 50 to create a mean score of the weight stigma frequency. Average scores range from 0 to 3, with higher scores indicating a higher frequency of experiencing weight stigma. The Cronbach’s alpha for the overall inventory was 0.94 in the present study.

#### 2.3.4. Binge Eating

Binge eating was measured using the Binge Eating Scale [47]. The scale contains 16 items, every item has three or four statements, and each statement is independently scored from 0 to 3 or 0 to 4.

Participants can only choose one statement for each item. A sample item with three statements includes “(1) I don’t feel any guilt or self-hate after I overeat (score = 0); (2) After I overeat, occasionally I feel guilt or self-hate (score = 1); and (3) Almost all the time I experience strong guilt or self-hate after I overeat (score = 3)”. The overall scale score ranges from 0 to 46. Higher scores suggest a greater degree of binge eating severity. The Binge Eating Scale with different language versions has been validated in Asian populations with a Cronbach’s alpha range from 0.85–0.86 [44,48]. The English version of the Binge Eating Scale was used in the present study, and the Cronbach’s alpha for the overall scale was 0.86 in our sample. 

#### 2.3.5. Level of Acculturation

The level of acculturation of the participants was measured using the Suinn-Lew Asian Self-Identity Acculturation Scale [49]. The scale is the most often used acculturation scale for Asian Americans [50]. The scale contains 21 items with a 5-point scale (1 = low acculturation, 3 = bicultural, 5 = high acculturation) to measure both actual behaviors and assessed ideals or preferences. The overall scale scores ranged from 1 (i.e., low acculturation, high Asian identity) to 5 (i.e., high acculturation, high Western identity); specifically, a score of 1 or 2 = Asian identity, a score of 3 = bicultural, and a score of 4 or 5 = Western-identified. In the present study, item 12 of the scale was used to measure the participants’ generation (1st generation = “I was born in Asia or a country other than the US”; 2nd generation = “I was born in the US, either parent was born in Asia or a country other than the US”; or “I don’t know what generation best fits me since I lack some information.” The Cronbach’s alpha for the overall scale was 0.86 in the present study.

#### 2.3.6. Level of Perceived Racism 

The Subtle and Blatant Racism Scale for Asian Americans was used to measure the level of perceived racism [51]. The purpose of the scale is to measure the extent to which an individual believes he or she has personally encountered racial discrimination. The scale contains 10 items with a 5-point scale (1 = almost never, 2 = once in a while, 3 = sometimes, 4 = often or frequent, 5 = almost always). Higher scores represent higher perceived personal experiences of racial discrimination. The Cronbach’s alpha for the overall scale was 0.79 in the present study. 

#### 2.3.7. Level of Perceived Stress 

The Perceived Stress Scale was used to measure the level of perceived stress [52]. The purpose of the scale is to measure the degree to which situations in an individual’s life are appraised as stressful and the perceptions of the individual’s capacity to manage perceived difficulties. The scale contains 10 items with a 5-point scale (0 = never, 1 = almost never, 2 = sometimes, 3 = fairly often, 4 = very often). Scores range from 0 to 40, with higher composite scores indicative of greater perceived stress. The Cronbach’s alpha for the overall scale was 0.75 in the present study.

### 2.4. Procedure

The participants were recruited through advertisements and flyers from local communities, Chinese language schools, and churches. Potential participants who were interested in the study were screened over the phone for eligibility. Eligible participants were then invited to a Biobehavioral Laboratory for measuring heights and weights in a private room before enrollment. The purpose of the study, the procedures, and the potential benefits and harms were fully explained to the eligible participants if the study eligibility criteria were met. The eligible participants were told that they could choose not to participate in this study and could freely withdraw from the study at any time without negative consequences. After all the questions related to the study were answered, the eligible participants were invited to sign the consent. After consent was obtained, the participants completed paper questionnaires in the same private room and received a $30 gift card. 

### 2.5. Statistical Analyses

The mean, standard deviation, and range or frequency and percentage, as appropriate, were used to describe the demographic variables of height, weight, and BMI and the scores of all five questionnaires. Independent-groups two-sample *t*-tests were applied to compare the mean scores between the different levels of weight status (i.e., “overweight” versus “obese”) separately for each of the five questionnaires. A Pearson correlation analysis was used to determine the bivariate relationships between the pairs of continuous variables.

Multiple regression analyses were used to determine the relationship between the experienced weight stigma (as the independent variable) and binge eating (as the dependent variable). For Model 1 (*n* = 166), we adjusted for age, BMI, the number of years lived in the US, the level of perceived racism for Asian Americans, and the level of perceived stress as covariates, because those variables were significantly correlated with the frequency of weight stigma or binge eating. We also included sex, education, and immigration generation (1st versus 2nd generation) as additional covariates. Shapiro-Wilk, Kolmogorov-Smirnov, Cramer-von Mises, and Anderson-Darling statistics were performed to assess the assumption of normally distributed residuals. Residual-predicted value plots were generated to determine the homogeneity of the variances for all models. The results of the diagnostic tests showed that the assumptions of normality and homogeneity of variance were violated in Model 1. We examined the model distributions and probability plots for the studentized residuals, and removed the observations corresponding to the seven highest values and the one lowest studentized residual value of binge eating. We then subsequently refit the model (*n* = 158) with the same variables but without these eight outliers to improve the model diagnostics. 

To analyze whether the level of acculturation moderates the relationship between experienced weight stigma and binge eating, we entered the level of acculturation into the multiple regression specified above as Model 1 (*n* = 166) as a moderator through its main effect and pairwise interaction with the frequency of weight stigma to produce Model 2. We assessed whether the level of acculturation is a significant moderator by examining whether the interaction term was statistically significant. We adjusted for age, BMI, the number of years lived in Asia and the US, the level of perceived racism for Asian Americans, and the level of perceived stress as covariates in Model 2, because these variables were significantly correlated with experienced weight stigma, binge eating, or acculturation. Again, sex, education, and immigration generation (1st versus 2nd generation) were included as additional covariates. We additionally conducted another multiple linear regression analysis for which we removed the covariates of years lived in Asia and the US from Model 2, because these two variables were highly correlated with acculturation and may introduce collinearity. The violation of the normality assumption was again found for Model 2; therefore, we removed the same eight outliers of studentized residual values of binge eating from the analyses and re-fitted the model to obtain more well-behaved model diagnostics. SAS 9.4 software was used for all the data analyses [53]. Two-sided *p*-values of 0.05 or less were considered statistically significant, with no adjustment for multiple testing in the present study. 

## 3. Results

### 3.1. Participant Characteristics

Table 1 presents the results of the characteristics of all the participants. The study sample included 166 adults (92 men, 74 women) who had a mean (±standard deviation) age of 45.7 ± 9.8 years and a mean BMI of 26.6 ± 3.1 kg/m^2^ (68.7% “overweight” and 31.3% “obese”). The majority of the participants identified as first-generation Asian Americans (94%) with graduate degrees (70.5%). The average years lived in Asia were 26.6 ± 9.4 years compared with 18.6 ± 9.8 years in the US.

### 3.2. Descriptive Statistics and Correlations

The results of the descriptive statistics and Pearson correlations are shown in Table 2 and Table 3, respectively. A majority (89.8%) of the participants had experienced weight stigma in their lifetime (Stigmatizing Situations Inventory score greater than 0). The overall mean score for the experienced weight stigma and binge eating of all participants was low, and the participants who were in the “obese” category reported more weight stigma experiences and greater binge eating than those in the “overweight” category. The mean score of the Suinn-Lew Asian Self-Identity Acculturation Scale was 2.21 ± 0.42 for all the participants, meaning that the participants tended to identify themselves as having an “Asian identity” (score of 1 or 2) rather than being “Western identified” (score of 4 or 5). Additionally, 94% of the participants (*n* = 156) scored 1 or 2 on the Acculturation Scale, indicating that they retained identity with their ethnic heritage as Asians. There were no significant differences between the weight categories with respect to the mean acculturation levels. Low levels of perceived racism and stress were observed in our sample. Experienced weight stigma was positively correlated with binge eating and acculturation. Age, BMI, the number of years lived in Asia, the number of years lived in the US, the level of perceived racism, and the level of perceived stress were all significantly correlated with acculturation, but only some of the variables significantly correlated with experienced weight stigma and binge eating.

### 3.3. Association between Experienced Weight Stigma and Binge Eating

The refitting of Model 1 without outliers had a minimal effect on the results, therefore we present the results of Model 1 for all the participants (Table 4). In Model 1, the results indicated a significant association between the experienced weight stigma and binge eating after adjusting for age, BMI, number of years lived in the US, the level of perceived racism against Asian Americans, the level of perceived stress, sex, education, and immigration generation. The results illustrated that experienced weight stigma was a significant explanatory factor in binge eating. 

### 3.4. Moderator Role of Acculturation 

We present Model 2 for all the participants because the results of the refitting of Model 2 without outliers remained robust compared to the results including all the participants. In Model 2 (Table 5), the findings indicated that the interaction between the experienced weight stigma and acculturation was not a statistically significant effect for binge eating after adjustment for age, BMI, the number of years lived in Asia and the US, the perceived racism against Asian Americans, the perceived stress, sex, education, and immigration generation; therefore, we did not observe strong evidence that acculturation moderates the effect. The interaction remained nonsignificant when the covariates of the years lived in Asia and the US were removed from Model 2.

## 4. Discussion

The results of the current study show that the majority of the participants experienced weight stigma. Still, the mean score of experienced weight stigma was low, indicating that our participants reported that they did not often encounter weight stigma situations in their lifetime. Our result is similar to our first author’s previous Taiwanese weight stigma research in that the mean score of experienced weight stigma was low and the majority of the participants (78.7%) reported experiencing only one episode of weight stigma in their lifetime [44]. Although the Stigmatizing Situations Inventory is a well-known instrument to measure the frequency of weight stigma experiences, it requires participants to retrospectively recall the experiences of weight discrimination that happened in their lifetime. Thus, responder bias can happen due to poor or incomplete memory recall and lead to a low score of reported weight stigma [54]. Our result also illustrates that the participants in the “obese” BMI category experienced more weight stigma than the participants in the “overweight” BMI category, suggesting that persons with larger body sizes are more likely to become targets of weight discrimination [55]. However, it is worth mentioning that individuals who do not have larger body sizes may still face weight stigma situations. For example, the majority of our participants’ BMI is under 27.5 kg/m^2^, which may not be visually judged as having a larger body size. However, most of them were first-generation immigrants and still have close relationships with their family members who live in Asia, where lower BMI cut-off points for “overweight” (e.g., BMI ≥ 22 kg/m^2^) and “obesity” (e.g., BMI ≥ 26 kg/m^2^) are used [38]. Our participants might still receive criticism of their body sizes from their family members who live in Asia, given the relationship they have with their Asian families. 

Our findings are consistent with previous studies that binge eating was positively correlated with BMI [36], and the individuals who were in the “obese” category reported a greater degree of binge eating than those in the “overweight” category [56]. We also found that the degree of binge eating was low in this Asian American group. Given the evidence that a higher frequency of weight stigma experiences may be a risk factor for a greater degree of binge eating [14], the finding of the current study might be considered unsurprising, since the mean score of experienced weight stigma was low in our sample. Additionally, many studies illustrated that the higher the BMI, the greater the binge eating [36,57]. The fact that there were not as many “obese” participants in our sample may also explain the low degree of binge eating we observed in the present study. 

Our data showed that experienced weight stigma was uniquely associated with binge eating over and above other forms of stigmatization and stress, such as perceived racism and perceived stress. This result was consistent with Almeida, Savoy, and Boxer’s [58] study that weight stigma alone had a unique contribution to the prediction of binge eating after adjusting for other risk factors of binge eating such as depression, anxiety, and daily stress. Taken together, our findings, along with the previously noted study, illustrated the unique contribution of weight stigma on binge eating observed in not only the Western population but also in Asian Americans as well. Evidence from cohort studies suggested that individuals who experienced multiple forms of interpersonal discrimination or disadvantage were more likely to have worse health outcomes, such as depression and limitations in daily living activities, than others who experienced a single type of interpersonal discrimination or disadvantage [59]. Thus, it is essential to identify all possible forms of discrimination or disadvantage a person may have because interventions that target multiple forms of discrimination and disadvantage are more effective compared with targeting only a particular type of discrimination or disadvantage [60]. It should also be noted that weight-related self-stigma, or internalized weight stigma, may play a role in potential psychological mechanisms underpinning the connection between weight stigma and binge eating. The internalization of weight stigma (i.e., individuals’ belief that negative stereotypes about individuals with overweight and obesity apply to them) may occur after the direct or indirect experience of weight-related discrimination, and may mediate the association between stigmatizing experiences and binge eating [61]. Individuals with a high internalized weight bias believe that they should be stigmatized by others’ negative weight-related attitudes and comments, and such self-stigma may make the individuals vulnerable to unhealthy weight control practices, such as binge eating and purging [62]. Our results demonstrate that weight stigmatization explains additional variance in binge eating, even after controlling for the effects of racial discrimination and general stress. Future interventions for binge eating prevention in Asian Americans should consider weight stigmatization as an important risk factor. To date, research about the connections between weight stigma and binge eating have tended to be focused on mostly non-Hispanic white samples. Future research should include Asian Americans to investigate possible mechanisms between weight stigma and binge eating to reduce health disparities between ethnic groups in eating disorder diagnosis, treatment, and prevention.

Our results indicated that the level of acculturation did not significantly affect the relationship between the experienced weight stigma and binge eating after adjusting for the relevant covariates. This finding is consistent with Eisenberg et al. [33], which found that the associations between weight-based teasing and well-being (i.e., body satisfaction, self-esteem, and depression) among youth were similar across non-Hispanic white and other ethnic groups and not modified by the levels of acculturation among youths from immigrant communities in the US. There are two potential explanations for the nonsignificant moderating role of acculturation on the association between the experienced weight stigma and binge eating identified in the present study. First, the majority of our participants reported that they were of Asian identity, which suggests that our sample may not have represented a wide range of levels of acculturation. It was difficult to determine whether the level of acculturation affected the relationship between the experienced weight stigma and binge eating, since our sample represented only a certain degree of acculturation. Second, we acknowledge that the statistical power for testing interactions was lower than that for testing the main effects. The lack of adequate statistical power in the present study may contribute to the nonsignificant moderating role of acculturation between the experienced weight stigma and binge eating. The effect size of 0.12 that we used in the sample size estimation was from a hierarchical regression analysis of binge eating with both weight stigma and BMI as explanatory factors in the model [44]. The present study might be underpowered to detect a moderation effect when using an effect size of 0.12 (When we attempted to retrieve the data used in the 2015 APJCN paper [44] and tried to test the unique contribution of the variance of binge eating explained by weight stigma, we discovered that a portion of the data was not useable due to unknown computer issues). A larger sample size might have provided adequate power to detect a statistically significant positive effect of acculturation on the relationship between experienced weight stigma and binge eating. 

Several limitations are worth noting. First, we were not able to conclude temporal causality, since the exposure (i.e., weight stigma) and outcome (i.e., binge eating) were simultaneously measured [63]. Second, the findings cannot be assumed to be generalizable to the whole Asian American population, since our sample was almost exclusively Chinese-speaking (93%) and first-generation (94%) Asian Americans. Third, the average experience of weight stigma and binge eating were low, indicating that this Asian American sample might not have been the best group to study this topic. Moreover, our findings cannot speak to clinical levels of binge eating, since the Binge Eating Scale [48] is not a clinical diagnostic tool and can only indicate the tendency toward binge eating. 

Future research can consider recruiting clinical-based samples such as Asian American patients with BED from eating disorder clinics, or Asian American patients from weight loss centers, to assess better the relationship between weight stigma and binge eating for Asian Americans in higher-risk groups. Future studies should also reach out to diverse ethnic groups of Asian Americans (e.g., Vietnamese, Japanese, and Filipino Americans), and these should span multiple generations to explore the role of acculturation further. Finally, weight stigma researchers should use a longitudinal study design in the future to further understand the causal relationship between weight stigma and binge eating and the possible long-term impact of weight stigma on health outcomes in Asian American populations.

## 5. Conclusions

This study sheds light on the relationship between experienced weight stigma and binge eating in Asian Americans, who are an underrepresented population in the many fields of research. The findings highlight that a higher frequency of experiencing weight stigma is associated with a greater degree of binge eating, and the influence of experienced weight stigma on binge eating is above and beyond other types of discrimination or stressors, such as racism and general stress. We did not find significant evidence of moderation by acculturation. The firmly established relationship between weight stigma and binge eating from the previous and present studies suggest that research in the fields of weight stigma and binge eating should include Asian Americans to reduce racial and ethnic health disparities.

## Figures and Tables

**Table 1:** Demographic characteristics of participants (*n* = 166).

Characteristics/Categories	Mean (SD)/*n* (%)	Range
Age	45.7 (9.8)	21–65
Male	92 (55.4%)	
Female	74 (44.6%)	
Body height (centimeter)	166.9 (8.7)	149.5–191.0
Body weight (kilogram)	74.4 (12.3)	52.7–120.1
BMI (kg/m^2^)	26.6 (3.1)	23.0–45.3
Overweight (23.0–27.5 kg/m^2^)	114 (68.7%)	
Obesity (>27.5 kg/m^2^)	52 (31.3%)	
Education		
Graduate school	117 (70.5%)	
College/university or below	49 (29.5%)	
Generation		
1st Generation	156 (94.0%)	
2nd Generation	9 (5.4%)	
Don’t know	1 (0.6%)	
Number of years lived in Asia	26.6 (9.4)	0–60
Number of years lived in the US	18.6 (9.8)	1–44

1st generation = participant was born in Asia or country other than the US; 2nd generation = participant was born in the US, and either parent was born in Asia or country other than US; SD = standard deviation; *n* = number of participants; % = percentage of participants.

**Table 2:** Descriptive statistics for experienced weight stigma, binge eating, acculturation, perceived racism, and perceived stress by the total sample and weight status.

Variables	Total Group (*n* = 166)	Overweight (*n* = 114)	Obese (*n* = 52)	*t*	*p*-Value
	Mean ± SD	Mean ± SD	Mean ± SD		
Experienced weight stigma	0.28 ± 0.33	0.22 ± 0.30	0.40 ± 0.36	3.28	0.0013
Binge eating	8.25 ± 6.88	7.54 ± 6.76	9.83 ± 6.94	2.01	0.0462
Acculturation	2.21 ± 0.42	2.20 ± 0.36	2.24 ± 0.51	0.55	0.5859
Perceived racism	20.07 ± 5.68	20.44 ± 5.02	19.25 ± 6.88	−1.12	0.2672
Perceived stress	16.20 ± 5.62	15.82 ± 5.42	17.04 ± 6.03	−0.42	0.1948

“Overweight” = BMI: 23.0–27.5 kg/m^2^; “Obese” = BMI: >27.5 kg/m^2^; SD = standard deviation; *n* = number of participants; *t* = independent groups *t*-test of difference weight status (degrees of freedom = 164).

**Table 3:** Pearson correlation coefficients between pairs of study variables (*n* = 166).

Measures	1	2	3	4	5	6	7	8
1. Age	-							
2. BMI	0.02							
3. Years lived in Asia	0.47 **	−0.11						
4. Years lived in the US	0.51 **	0.13	−0.46 **					
5. Experienced weight stigma	−0.18 *	0.40 **	−0.12	−0.06				
6. Binge eating	−0.26 **	0.28 **	−0.20 *	−0.09	0.70 **			
7. Acculturation	−0.29 **	0.23 **	−0.66 **	0.33 **	0.27 **	0.26 **		
8. Perceived racism	−0.17 *	0.01	−0.14	−0.04	0.41 **	0.42 **	0.19 *	
9. Perceived stress	−0.22 **	0.12	−0.06	−0.16 *	0.30 **	0.27 **	0.17 *	0.31 **

* *p* < 0.05; ** *p* < 0.01.

**Table 4:** Results from the multiple linear regression for the association of experienced weight stigma with binge eating.

Parameters	Model 1 (*n* = 166)			
	Binge Eating			
	B	β	(95% CI)	*R* ^2^
				0.54 **
Experienced weight stigma	12.25	0.58	(9.34, 15.15) **	
Age (year)	−0.06	−0.08	(−0.16, 0.05)	
BMI (kg/m^2^)	0.11	0.05	(−0.17, 0.39)	
Years lived in the US	−0.02	−0.03	(−0.11, 0.08)	
Perceived racism	0.17	0.14	(0.02, 0.33) *	
Perceived stress	0.01	0.01	(−0.13, 0.16)	
Sex	−0.57	−0.04	(−2.17, 1.03)	
Education	1.39	0.09	(−0.31, 3.10)	
Generation	−2.50	−0.09	(−6.44, 1.45)	

Generation = immigration generation, B = unstandardized parameter estimate; **β** = standardized parameter estimate; 95% CI = 95% confidence interval; *R^2^* = coefficient of determination; * *p* < 0.05; ** *p* < 0.01.

**Table 5:** Results from a multiple linear regression for the moderating role of acculturation between the experienced weight stigma and binge eating.

Parameters	Model 2 (*n* = 166)			
	Binge Eating			
	B	β	(95% CI)	*R* ^2^
				0.54 **
Experienced weight stigma	8.41	0.40	(−0.40, 17.23)	
Acculturation	−0.73	−0.04	(−3.41, 1.94)	
Weight stigma × Acculturation	1.44	0.21	(−1.65, 4.54)	
Age (year)	−0.07	−0.11	(−0.16, 0.01)	
BMI (kg/m^2^)	0.11	0.05	(−0.18, 0.39)	
Perceived racism	0.18	0.15	(0.02, 0.33) *	
Perceived stress	0.02	0.02	(−0.12, 0.16)	
Sex	−0.50	−0.04	(−2.11, 1.11)	
Education	1.35	0.09	(−0.37, 3.06)	
Generation	−1.86	−0.06	(−6.15, 2.43)	

*p*-value for the interaction term of weight stigma × acculturation in Model 2 is 0.36; Generation = immigration generation, B = unstandardized parameter estimate; β = standardized parameter estimate; 95% CI = 95% confidence interval; *R*^2^ = coefficient of determination; * *p* < 0.05; ** *p* < 0.01.
